# Supplementary figures and images for: Isolation of the side population from neurogenic niches enriches for endothelial cells
Source: PLoS One. 2022 Jan 19;17(1):e0250752. doi: 10.1371/journal.pone.0250752 (PMC8769340; doi:10.1371/journal.pone.0250752)

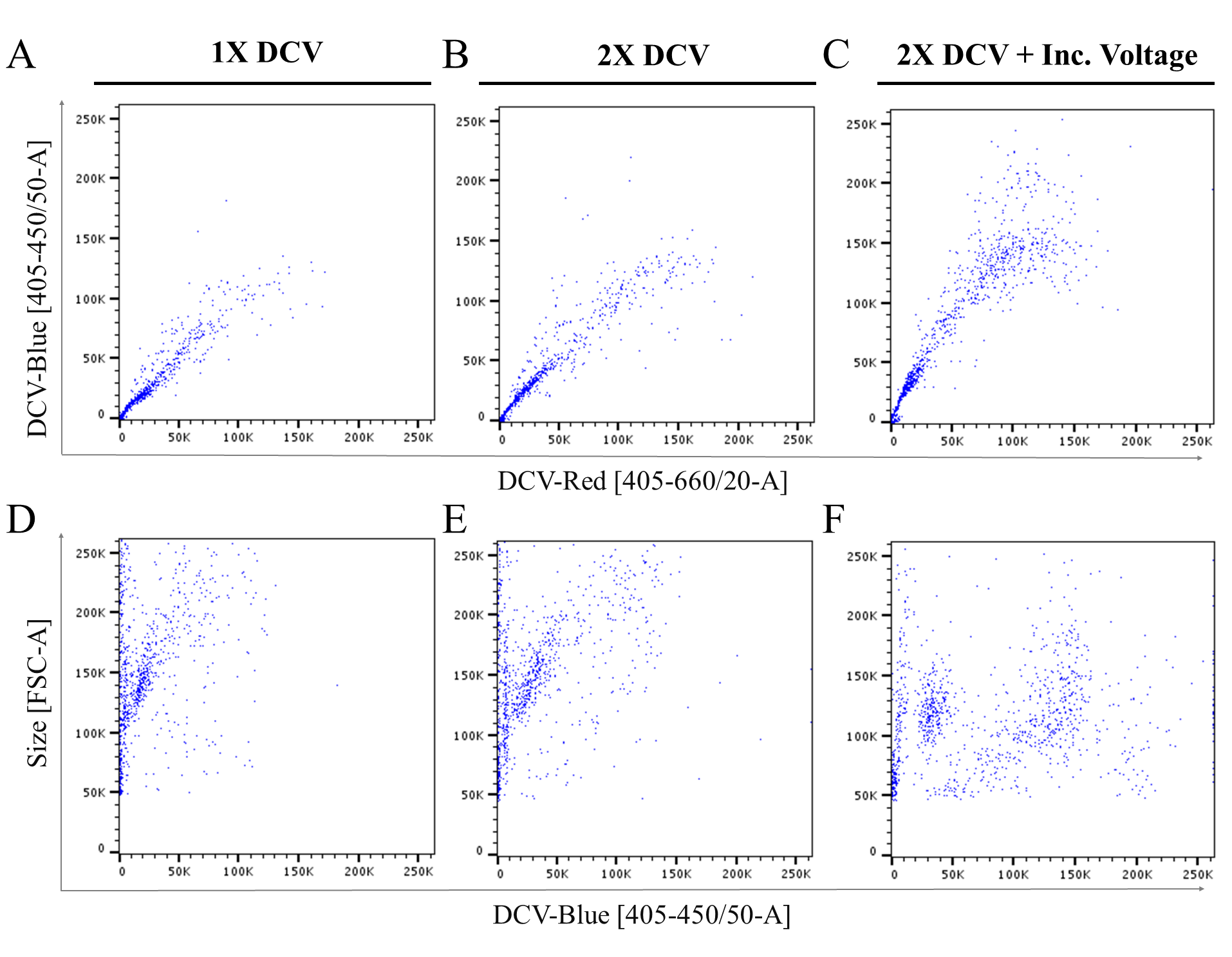

Supplement: S1 Fig — DCV staining testing 1X (A, D) or 2X DCV (B, E, C, F), as well as varying degrees of voltage for the 2X DCV concentration (B, E vs. C, F) as shown in dual fluorescence plots (A-C) and DCV-Blue/size plots (D-F). These results suggested 2X DCV with optimal excitation (C, F) was sufficient to distinguish the heterogeneous populations. Plots A, B, D, and E were generated from samples of pooled nine female mice. Plot C and F were generated from pooled samples of five male and three female mice, 2.5k cells are shown. (TIF) [file pone.0250752.s001.TIF]

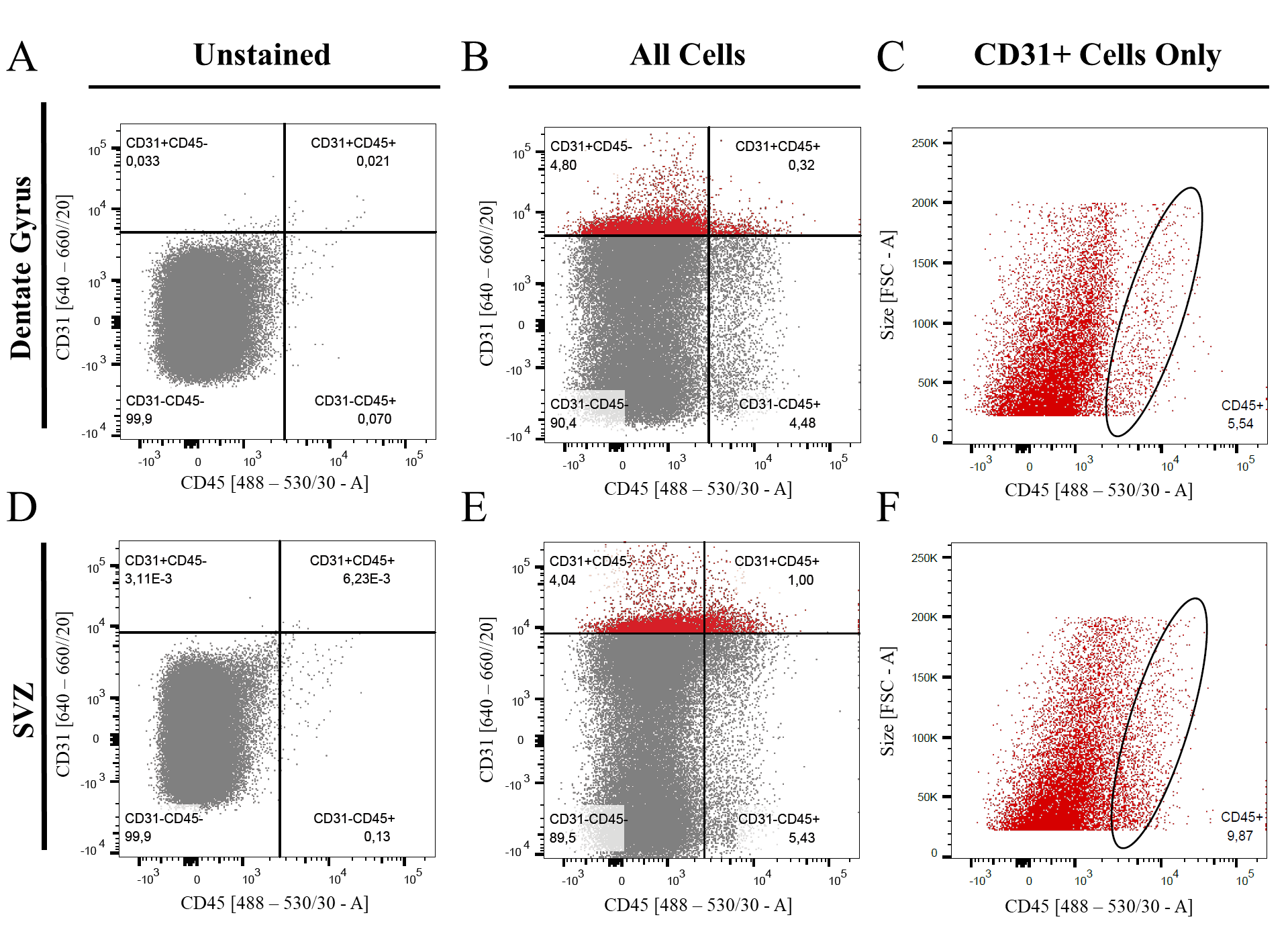

Supplement: S2 Fig — CD31 and CD45 staining in the dentate gyrus (A—unstained, B—all-stained) and the SVZ (D—unstained, E—all-stained) show little co-expression of CD31 and CD45 in the main populations (B and E, respectively) with only a small proportion of CD31+ cells expressing CD45 (C and F). These plots are generated based on pooled samples from five male and three female mice, 50k cells are shown. (TIF) [file pone.0250752.s002.TIF]
